# Supplementary material for: CaMKIIa+ neurons in the bed nucleus of the stria terminalis modulate pace of natural reward seeking depending on internal state
Source: Psychopharmacology (Berl). 2024 Feb 24;241(6):1245–63. doi: 10.1007/s00213-024-06561-5 (PMC11106149; doi:10.1007/s00213-024-06561-5)
Supplement: Supplementary file 1 — Supplementary Material 1 [file 213_2024_6561_MOESM1_ESM.pdf]

Supplemental information

CaMKIIa+ neurons in the bed nucleus of the stria terminalis modulate pace of natural reward seeking depending on internal state

Patty T. Huijgens[1,2,3], Roy Heijkoop[1], Louk J.M.J. Vanderschuren[2], Heidi M.B. Lesscher[2], Eelke M.S. Snoeren[1,3]

1. Department of Psychology, Faculty of Health Sciences, UiT The Arctic University of Norway, Tromsø, Norway
2. Department of Population Health Sciences, Section Animals in Science and Society, Faculty of Veterinary Medicine, Utrecht University, Utrecht, The Netherlands
3. Communicating authors: Eelke M.S. Snoeren, eelke.snoeren@uit.no; Patty T. Huijgens, patty.t.huijgens@uit.no

Supplemental Figure 1

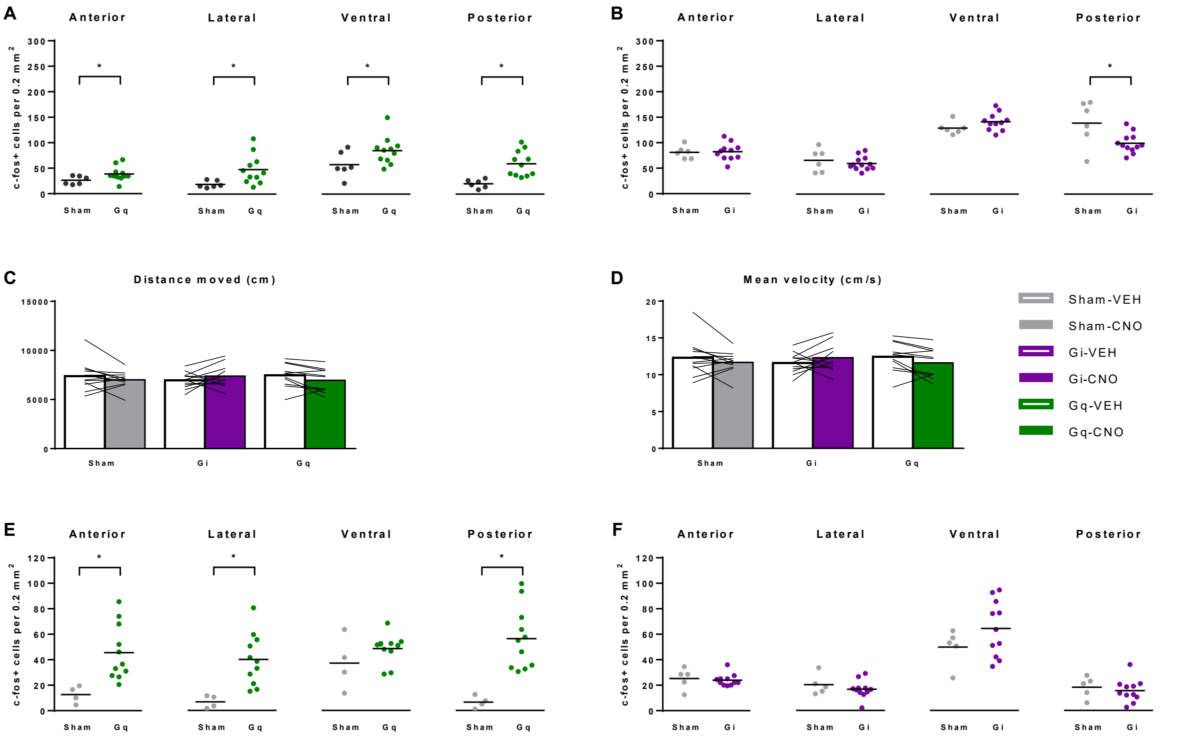

***Supplementary Figure 1. Effects of CNO treatment on c-Fos+ cell count and locomotor activity (A)** Number of c-Fos+ cells after CNO injection of Sham (n=6) or Gq-rats (n=11) in homecage of the sexual behavior experiment. (The Sham rats are the same rats as Fig. 1D-HC) **(B)** Number of c-Fos+ cells after CNO injection of Sham (n=6) or Gi-rats (n=11) after copulation to one ejaculation of the sexual behavior experiment. (The Sham rats are the same rats as Fig. 1D-HC) **(C)** Total distance moved during SIM test. n = 12 for Sham and Gi bars, n = 11 for Gq bars. **(D)** mean velocity during the SIM test. n = 12 for Sham and Gi bars, n = 11 for Gq bars. **(E)** Number of c-Fos+ cells CNO injection of Sham (n=4) or Gq-rats (n=11) in homecage of the sucrose experiment. (The Sham rats are the same rats as Fig. 3D-HC) **(F)** Number of c-Fos+ cells after CNO injection of Sham (n=5) or Gi-rats (n=11) after 30 minutes of FR1 engagement the sucrose experiment. (The Sham rats are the same rats as Fig. 3D-FR1) **(All panels)** *p < 0.05.*

Supplemental Figure 2

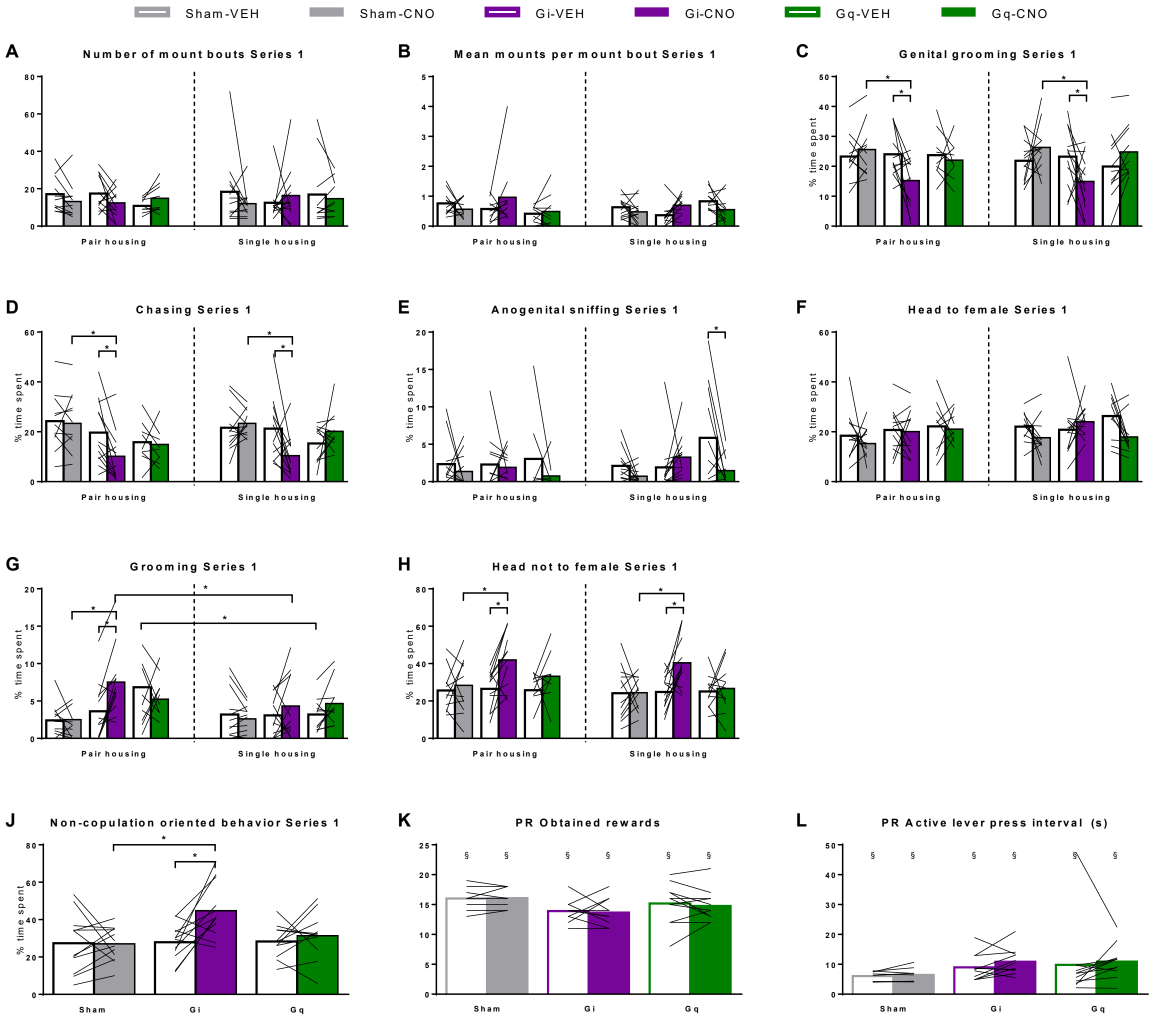

***Supplementary Figure 2. Effects of DREADD on copulation parameters of pair or single housed rats. (A)** Number of mount bouts preceding the first ejaculation (Series 1). **(B)** Mean number of mounts in a mount bout in the first ejaculation series. **(C)** Percentage of total time before the first ejaculation spent on genital grooming, **(D)** chasing, **(E)** anogenital sniffing, **(F)** having head in the direction of the female, **(G)** grooming of other regions than the genitals, and **(H)** Having head not in the direction of the female. **(All panels)** Left to right: pair housing n = 11, 12, 12, 12, 10, 10; single housing n = 12, 12, 12, 12, 11, 11. *p < 0.05. Statistical details of pair housing can be found in manuscript.*

Supplemental Figure 3

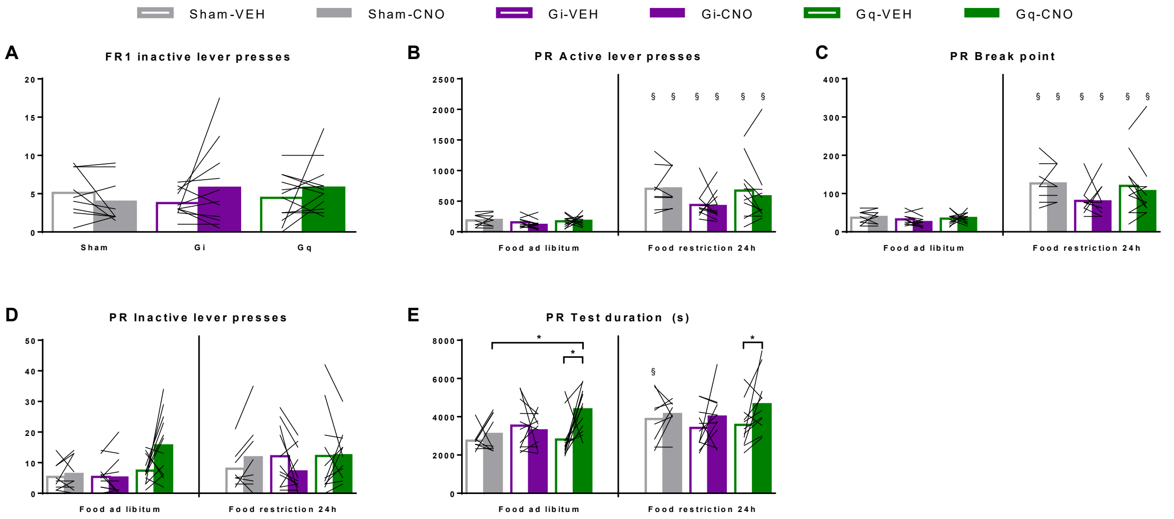

Supplementary Figure 3. Effects of DREADD on FR1 and PR sucrose self-administration with food available ad libitum (FR1 & PR) or after 24 hours of food restriction (PR). (A) *Number of inactive lever presses under FR1 in the total 30 minute test. n = 9 for Sham, 11 for Gi, 12 for Gq. Data points are averages of 2 vehicle- and 2 CNO-tests.* ***(B)*** *Total number of active lever presses during the PR test. §p < 0.05 compared to food ad libitum condition.* ***(C)*** *Break point defined by the maximum number of lever presses made to earn the last reward.* ***(D)*** *Number of inactive lever presses under PR.* ***(E)*** *Total duration of the PR test (test ends after 30 minutes of no rewards obtained).* ***(B, C, D, E)*** *Ad libitum food n = 9 for Sham, 11 for Gi, 12 for Gq; Food restriction n = 8 for Sham, 11 for Gi, 12 for Gq. *p <0.05. Statistical details not mentioned here can be found in manuscript.*

Supplemental Figure 4

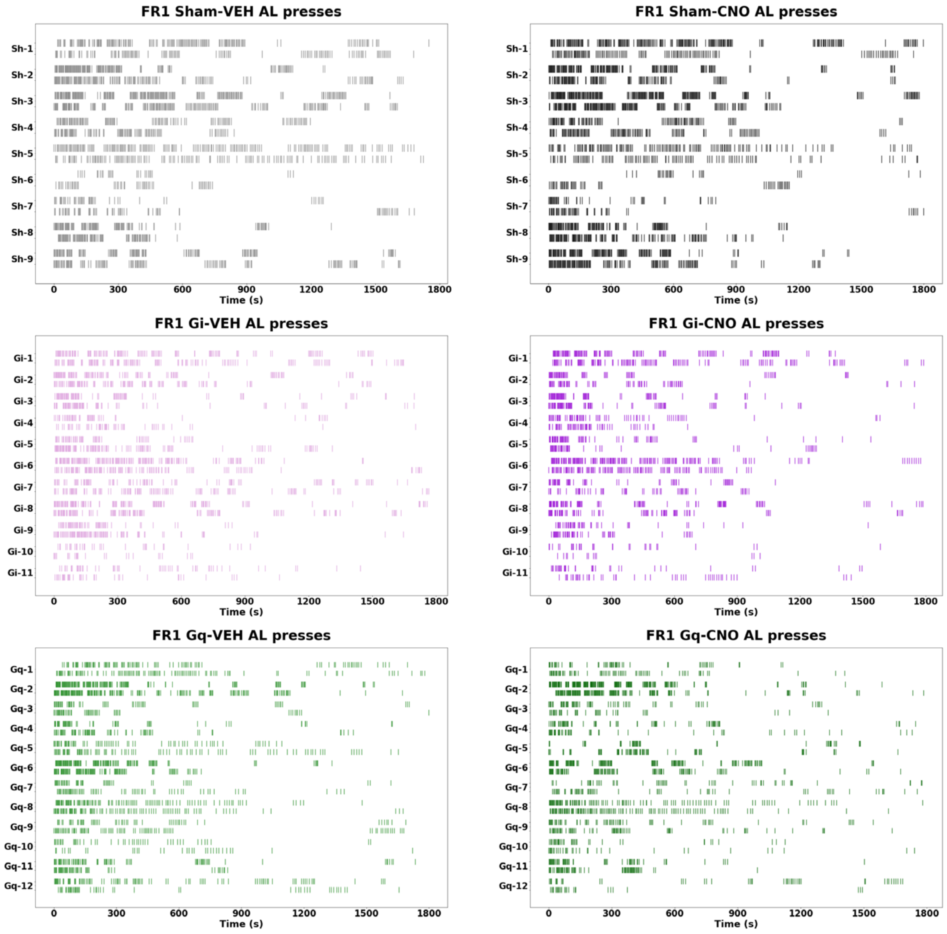

Supplementary Figure 4. *Event plots of FR1 active lever presses. Every line represents an active lever press timestamp. Each animal was tested twice after vehicle and twice after CNO and these tests are plotted separately.*

Supplemental Figure 5

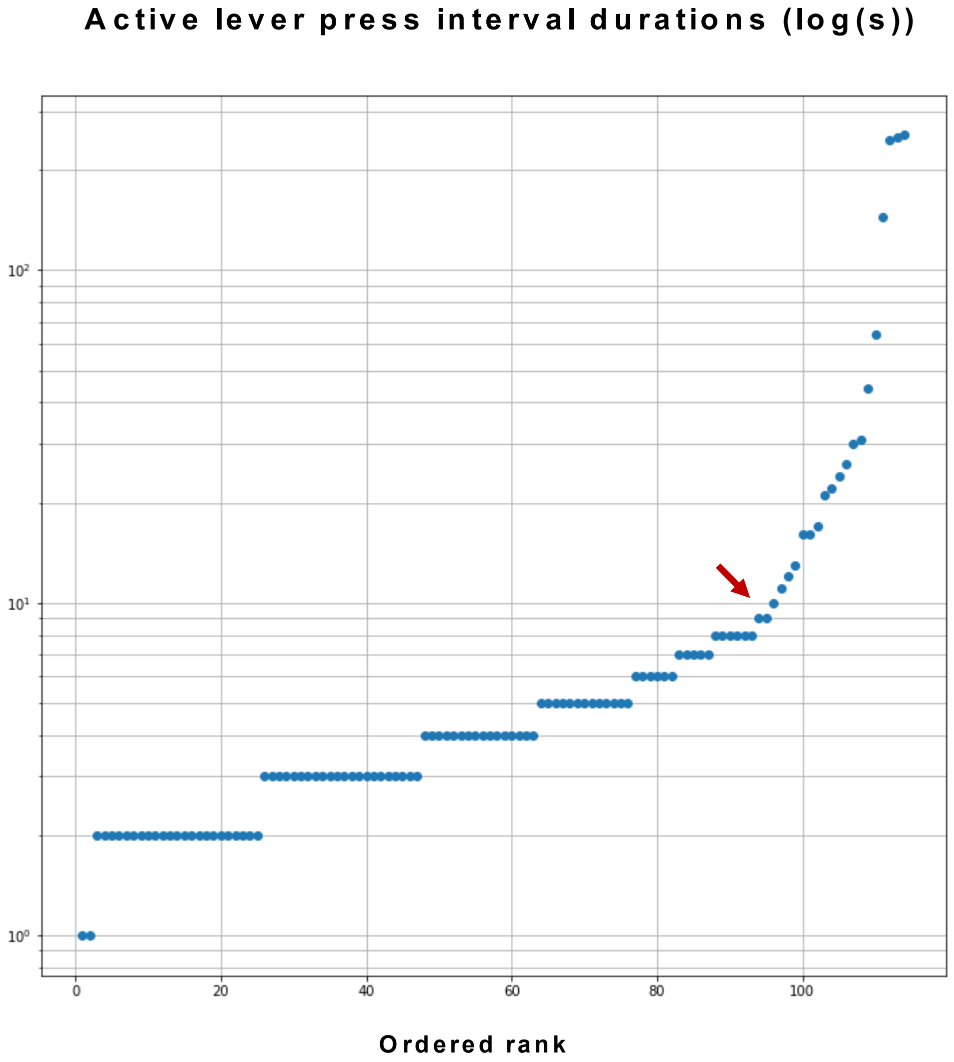

Supplementary Figure 5. *Scree plot example with indication of inflection point estimation (red arrow).*

Supplemental Figure 6

A

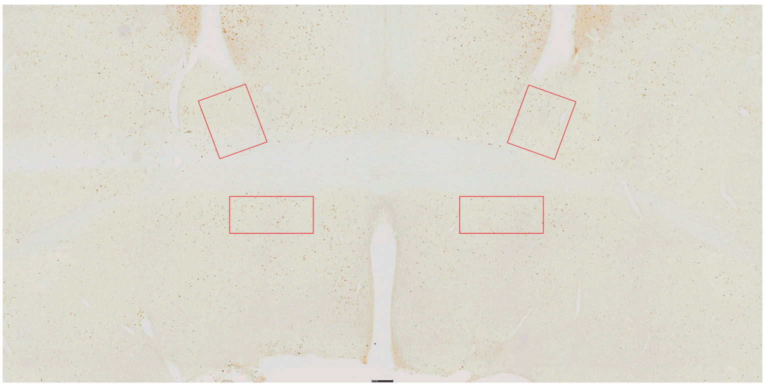

B

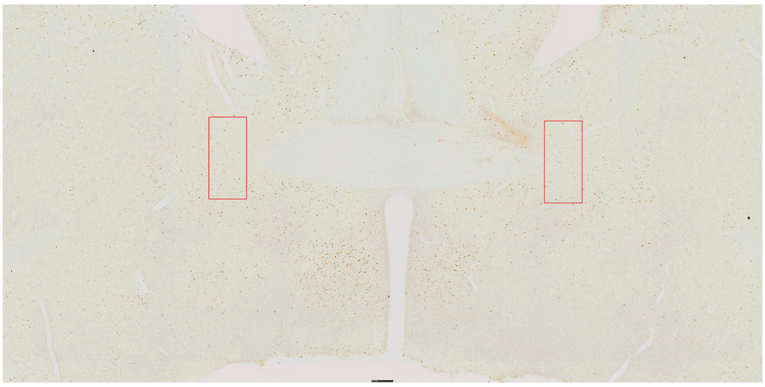

C

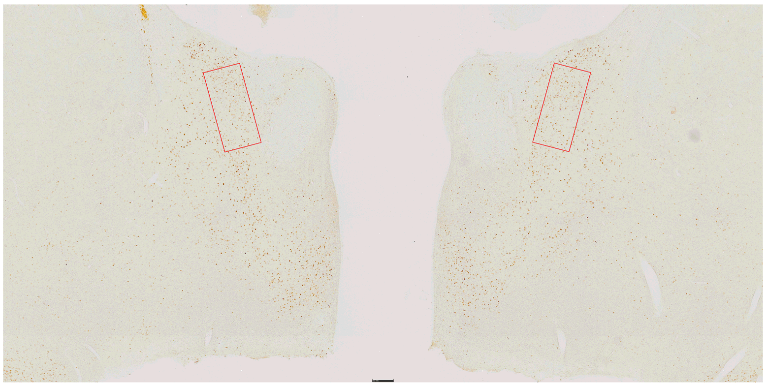

Supplementary Figure 6. *Example images with ROI counting boxes locations on c-Fos stained sections. (A) Anterior (top boxes) and ventral (bottom boxes) BNST ROI's (AP between 0.0mm and -0.12mm). (B) Lateral BNST ROI's (AP between -0.24mm and -0.36mm). (C) Posterior BNST ROI's (AP between -0.72mm and -0.9mm).*

Supplemental Figure 7

A

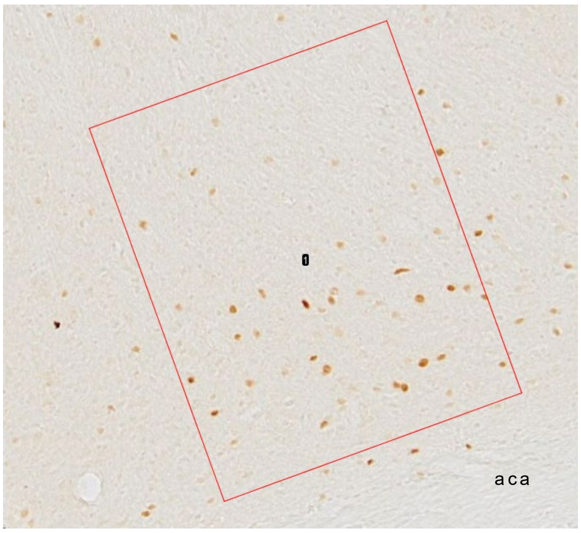

B

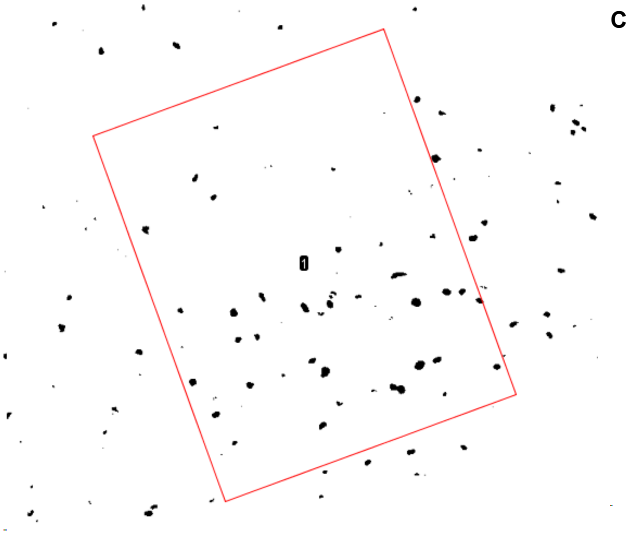

C

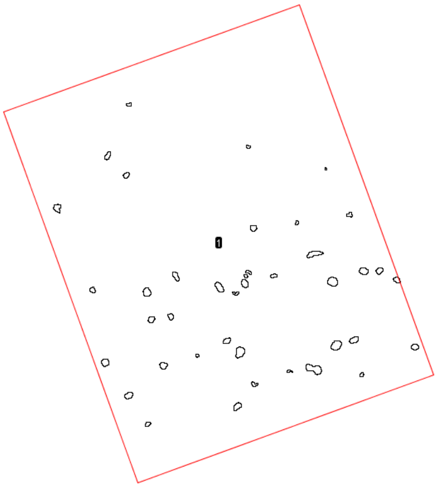

Supplementary Figure 7 *ImageJ c-Fos automated counting example. (A) Original image with a boxed area in the anterior BNST with c-Fos+ cells. aca; anterior commissure (B) Same image after thresholding and converting to binary. (C) Map of identified c-Fos+ cells by the particle analyze.*

Table S1. Statistical analysis, related to Figures 1-4, S1-S3.

Figure	N per group	Statistical test	Test and p-values	Post hoc test
1D	N=6	One-tailed independent samples t-test	anterior t(10)=9.09, p<0.001; ventral t(10)=6.19, p<0.001	
		One tailed Mann-Whitney U test	lateral U=0, p=0.001; posterior U=0, p=0.001	
1E	Sham N=12<br>Gi N=11<br>Gq N=12	Paired t-test - time in female vs male zone	VEH: Sham t(11)=6.19, p<0.001; Gi t(11)=6.83, p<0.001; Gq t(10)=10.46, p<0.001<br>CNO: Gi t(11)=5.58, p<0.001<br>Gq t(10)=3.69, p=0.004	
		Linear mixed model: virus*treatment	F(5, 63)= 4.69, p<0.001	Gi: VEH-CNO p=0.007<br>Sham: VEH-CNO p=0.028
1F	Sham N=12<br>Gi N=11<br>Gq N=12	One-sample t-test compared to 0.5	VEH: Sham t(11)=6.34, p<0.001; Gi t(11)=9.24, p<0.001; Gq t(10)=12.76, p<0.001<br>CNO: Gi t(11)=6.29, p<0.001<br>Gq t(10)=3.84, p=0.003	
		Linear mixed model: virus*treatment	F(5, 63)= 4.69, p<0.001	Gi: VEH-CNO p=0.054<br>Gq: VEH-CNO p=0.036
1G	Sham N=12<br>Gi N=11<br>Gq N=12	Linear mixed model: virus*treatment	F(5,69)=7.12,p=0.023	Gi: VEH-CNO p=0.023<br>CNO: Gi-Sham p=0.019
2B	Sham N=11 (left) and N=12 (right)<br>Gi N=12<br>Gq N=10	Linear mixed model: virus*treatment	F(5,69)=4.26, p=0.002	Gi: VEH-CNO p=0.035<br>CNO: Gi-Sham p=0.009
2C	Sham N=11 (left) and N=12 (right)<br>Gi N=12<br>Gq N=10	Linear mixed model: virus*treatment	F(5,70)=3.04, p=0.015	CNO: Gi-Sham p=0.020
2D	Sham N=11 (left) and N=12 (right)<br>Gi N=12<br>Gq N=10	Linear mixed model: virus*treatment	NS	
2E	Sham N=11 (left) and N=12 (right)<br>Gi N=12<br>Gq N=10	Linear mixed model: virus*treatment	NS	

2F	Sham N=11 (left) and N=12 (right)<br>Gi N=12<br>Gq N=10	Linear mixed model: virus*treatment	NS	
2G	Sham N=11 (left) and N=12 (right)<br>Gi N=12<br>Gq N=10	Linear mixed model: virus*treatment	$F(5,66)=3.83$, $p<0.004$	Gi: VEH-CNO $p=0.018$
2H	Sham N=11 (left) and N=12 (right)<br>Gi N=12<br>Gq N=10	Linear mixed model: virus*treatment	$F(5,53)=2.79$, $p=0.026$	Gi: VEH-CNO $p<0.001$<br>CNO: Gi-Sham $p<0.001$
2I	Sham N=10 (left) and N=11 (right)<br>Gi N=11 (left) and N=8 (right)<br>Gq N=10	Linear mixed model: virus*treatment	$F(5,68)=3.58$, $p=0.006$	Gi: VEH-CNO $p=0.002$<br>CNO: Gi-Sham $p=0.008$
2J	Sham N=11 (left) and N=12 (right)<br>Gi N=12<br>Gq N=10	Linear mixed model: virus*treatment	$F(5,62)=8.96$, $p<0.001$	Gi: VEH-CNO $p<0.001$<br>CNO: Gi-Sham $p<0.001$
3B	HC, N=4,<br>FR1 N=5	One-tailed independent samples t-test	Sham-FR1 vs. Sham-HC; anterior $t(7)=2.46$, $p<0.022$; lateral $t(7)=2.90$, $p=0.012$; posterior $t(7)=2.48$, $p=0.021$	
3C	Sham N=9<br>Gi N=11<br>Gq N=12	Linear mixed model: virus*treatment	$F(5,30)=4.14$, $p=0.006$	Gi: VEH-CNO $p=0.005$<br>CNO: Gi-Sham $p=0.017$<br>Gq-Sham $p=0.023$
3F	Sham N=9<br>Gi N=11<br>Gq N=12	Linear mixed model: virus*treatment	NS	
3G	Sham N=9<br>Gi N=11<br>Gq N=12	Linear mixed model: virus*treatment	NS	
3H	Sham N=9<br>Gi N=11<br>Gq N=12	Linear mixed model: virus*treatment	$F(5,33)=2.86$, $p=0.03$	Gi: VEH-CNO $p=0.023$
3I	Sham N=9<br>Gi N=11<br>Gq N=12	Linear mixed model: virus*treatment	NS	
3K	Sham N=9<br>Gi N=11	Linear mixed model: virus*treatment	$F(5,69)=3.18$, $p=0.012$	Gi: VEH-CNO $p=0.006$

	Gq N=12			CNO: Gi-Sham p<0.001<br>Gq-Sham p=0.036
4A	Sham N=12<br>Gi N=12<br>Gq N=10	Paired t-test - time in female vs male zone	VEH: Sham t(11)=7.98, p<0.001; Gi t(11)=9.07, p<0.001; Gq t(10)=6.95, p<0.001, and CNO: Sham t(11)=15.57, p<0.001; Gi t(11)=4.98, p<0.001; Gq t(10)=6.64, p<0.001.	
		Linear mixed model (=1E):<br>- virus*treatment<br><br>- virus*treatment*condition	 F(5, 63)= 4.69, p<0.001<br><br>NS	 Gi: VEH-CNO p=0.012
4B	Sham N=12<br>Gi N=12<br>Gq N=10	One-sample t-test compared to 0.5	VEH: Sham t(11)=10.82, p<0.001; Gi t(11)=12.22, p<0.001; Gq t(10)=6.98, p<0.001, and CNO: Sham t(11)=29.37, p<0.001; Gi t(11)=5.14, p<0.001; Gq t(10)=7.01, p<0.001.	
		Linear mixed model (=1F):<br>- virus*treatment<br><br>- virus*treatment*condition	 F(5, 68)= 3.53, p=0.007<br><br>NS	 Gi: VEH-CNO p=0.024
4C	Sham N=12<br>Gi N=12<br>Gq N=10	Linear mixed model (=1G):<br>- virus*treatment<br><br><br>- virus*treatment*condition	 F(5,69)=7.12, p=0.023<br><br><br>NS	 Gi: VEH-CNO p<0.001<br>CNO: Gi-Sham p<0.001
4D	Sham N=12<br>Gi N=12<br>Gq N=10	Linear mixed model (=2B):<br>- virus*treatment<br><br><br>- virus*treatment*condition	 F(5,69)=4.26, p=0.002<br><br><br>NS	 Gi: VEH-CNO p=0.006<br>CNO: Gi-Sham p=0.014
4E	Sham N=12<br>Gi N=12<br>Gq N=10	Linear mixed model (=2C):<br>- virus*treatment<br><br>- virus*treatment*condition	 F(5,70)=3.04, p=0.015<br><br>NS	 Gi: VEH-CNO p=0.034
4F	Sham N=12<br>Gi N=12<br>Gq N=10	Linear mixed model (=2H):<br>- virus*treatment<br>- virus*treatment*condition	 NS<br>NS	
4G	Sham N=12<br>Gi N=12<br>Gq N=10	Linear mixed model (=2I):<br>- virus*treatment<br><br>- virus*treatment*condition	 NS<br><br>F(5,67)=2.644, p=0.021	 <br><br>Pair-housing-Single housing: Gi-CNO p=0.002
4H	Sham N=12	Linear mixed model (=2F):		

	Gi N=12<br>Gq N=10	- virus*treatment<br><br>- virus*treatment*condition	F(5,67)=3.54, p=0.007<br><br>NS	Gi: VEH-CNO p=0.001
4I	Sham N=12<br>Gi N=12<br>Gq N=10	Linear mixed model (=2G):<br>- virus*treatment<br><br><br>- virus*treatment*condition	 F(5,66)=3.83, p<0.004<br><br><br>NS	 Gi: VEH-CNO p=0.002<br>CNO: Gi-Sham p=0.044
4J	Sham N=11 (left) and N=12 (right)<br>Gi N=11 (left) and N=8 (right)<br>Gq N=18 (left) and N=11 (right)	Linear mixed model (=2J):<br>- virus*treatment<br><br><br>- virus*treatment*condition	 F(5,62)=8.96, p<0.001<br><br><br>NS	 Gi: VEH-CNO p=0.001<br>CNO: Gi-Sham p=0.006
4K	Sham N=8<br>Gi N=11<br>Gq N=12	Linear mixed model (=3I):<br>- virus*treatment<br><br>- virus*treatment*condition	 NS<br><br>F(6,83)=24.90, p<0.001	 <br><br>*Ad libitum*-food restricted*:* p<0.001 across all groups (Sham, Gi, and Gq) and treatments)
4L	Sham N=8<br>Gi N=11<br>Gq N=12	Linear mixed model (=3K):<br>- virus*treatment<br><br>- virus*treatment*condition	 F(5,69)=3.18, p=0.012<br><br>F(6,84)=11.17, p<0.001	 NS<br><br>*Ad libitum*-food restricted*:* Gi-CNO p<0.001, Gi-VEH p<0.001, Gq-CNO p<0.001, Gq-VEH p=0.020, Sham-CNO p=0.033, Sham-VEH p=0.016
S1A	Sham N=6<br>Gq N=11	One-tailed independent samples t-test<br><br><br>One tailed Mann-Whitney U test	anterior t(15)=1.95, p=0.035; ventral t(15)=2.03, p=0.030<br><br>lateral U=9, p=0.007; posterior U=0, p<0.001	
S1B	Sham N=6<br>Gi N=11	One-tailed Mann-Whitney U test	posterior U=15, p=0.026	
S1C	Sham N=12<br>GI N=12<br>Gq N=11	Linear mixed model: virus*treatment	NS	
S1D	Sham N=12<br>GI N=12<br>Gq N=11	Linear mixed model: virus*treatment	NS	

S1E	Sham N=4<br>Gq N=11	One-tailed independent samples t-test<br><br>One-tailed Mann-Whitney U test	anterior t(13)=2.91, p=0.006<br><br>lateral U=0, p<0.001; posterior U=0, p<0.001	
S1F	Sham N=5<br>Gi N=11	One-tailed independent samples t-test or One-tailed Mann-Whitney U test	NS	
S2A	Pair housing:<br>Sham N=11<br>Gi N=12<br>Gq N=10<br>Single housing:<br>Sham N=12<br>Gi N=12<br>Gq N=11	Linear mixed model:<br>- virus*treatment<br>- virus*treatment*condition	 NS<br>NS	
S2B	Pair housing:<br>Sham N=11<br>Gi N=12<br>Gq N=10<br>Single housing:<br>Sham N=12<br>Gi N=12<br>Gq N=11	Linear mixed model:<br>- virus*treatment<br>- virus*treatment*condition	 NS<br>NS	
S2C	Pair housing:<br>Sham N=11<br>Gi N=12<br>Gq N=10<br>Single housing:<br>Sham N=12<br>Gi N=12<br>Gq N=11	Linear mixed model:<br>- virus*treatment<br><br>- virus*treatment*condition	 F(5,71)=4.59, p=0.001<br><br>NS	Pair housing:<br>Gi: VEH-CNO: p=0.011<br>CNO: Gi-Sham: p=0.10<br>Single housing: post-hoc vs VEH: p=0.019; post-hoc vs Sham-CNO:p=0.005
S2D	Pair housing:<br>Sham N=11<br>Gi N=12<br>Gq N=10<br>Single housing:<br>Sham N=12<br>Gi N=12<br>Gq N=11	Linear mixed model:<br>- virus*treatment<br><br>- virus*treatment*condition	 F(5,62)=7.22, p<0.001<br><br>NS	Gi: VEH-CNO: p=0.002<br>CNO: Gi-Sham: p=0.001
S2E	Pair housing:	Linear mixed model:<br>- virus*treatment	 F(5,68)=5.082, p=0.014	 Single housing:

	Sham N=11<br>Gi N=12<br>Gq N=10<br>Single housing:<br>Sham N=12<br>Gi N=12<br>Gq N=11	- virus*treatment*condition	NS	Gq: VEH-CNO: p=0.003
S2F	Pair housing:<br>Sham N=11<br>Gi N=12<br>Gq N=10<br>Single housing:<br>Sham N=12<br>Gi N=12<br>Gq N=11	Linear mixed model:<br>- virus*treatment<br>- virus*treatment*condition	NS<br>NS	
S2G	Pair housing:<br>Sham N=11<br>Gi N=12<br>Gq N=10<br>Single housing:<br>Sham N=12<br>Gi N=12<br>Gq N=11	Linear mixed model:<br>- virus*treatment<br>- virus*treatment*condition	F(5,55)=4.92, p<0.001<br>F(6,93)=2.74, p=0.017	Gi: VEH-CNO: p<0.001<br>CNO: Gi-Sham: p=0.002<br>Pair-housing-Single-housing: Gi p<0.001<br>Gq p=0.004
S2H	Pair housing:<br>Sham N=11<br>Gi N=12<br>Gq N=10<br>Single housing:<br>Sham N=12<br>Gi N=12<br>Gq N=11	Linear mixed model:<br>- virus*treatment<br>- virus*treatment*condition	F(5,65)=6.92, p<0.001<br>NS	Pair-housing:<br>Gi: VEH-CNO: p=0.001<br>CNO: Gi-Sham: p=0.013<br>Single housing:<br>Gi: VEH-CNO p=0.002<br>CNO: Gi-sham p=0.006
S3A	Sham N=9<br>GI N=11<br>Gq N=12	Linear mixed model:<br>virus*treatment	NS	
S3B	Ad libitum:<br>Sham N=9<br>Gi N=11<br>Gq N=12<br>Food restriction:<br>Sham N=8<br>Gi N=11<br>Gq N=12	Linear mixed model:<br>- virus*treatment<br>- virus*treatment*condition	NS<br>F(6,84)=14.72, p<0.001	*Ad libitum*-food restricted: Gi-CNO p=0.001; Gi-VEH p=0.002; p<0.001 across the other

				groups and treatments
S3C	Ad libitum:<br>Sham N=9<br>Gi N=11<br>Gq N=12<br>Food restriction:<br>Sham N=8<br>Gi N=11<br>Gq N=12	Linear mixed model:<br>- virus*treatment<br><br>- virus*treatment*condition	 NS<br><br>F(6,84)=17.240, p<0.001	 <br><br>*Ad libitum*-food restricted: Gi-VEH p=0.001; p<0.001 across the other groups and treatments
S3D	Ad libitum:<br>Sham N=9<br>Gi N=11<br>Gq N=12<br>Food restriction:<br>Sham N=8<br>Gi N=11<br>Gq N=12	Linear mixed model:<br>- virus*treatment<br>- virus*treatment*condition	 NS<br>NS	
S3E	Ad libitum:<br>Sham N=9<br>Gi N=11<br>Gq N=12<br>Food restriction:<br>Sham N=8<br>Gi N=11<br>Gq N=12	Linear mixed model:<br>- virus*treatment<br><br><br><br><br><br><br>- virus*treatment*condition	 F(5,68)=4.41, p=0.002<br><br><br><br><br><br><br>F(6,79)=2.62; p=0.046	 *Ad libitum:* Gq: VEH-CNO: p=0.002<br>CNO: Gq-Sham: p=0.026<br>Food restricted: Gq: VEH-CNO: p=0.019<br><br>*Ad libitum*-food restricted:<br>Sham-VEH p=0.040
